# Liraglutide, a once-daily human glucagon-like peptide 1 analogue, provides sustained improvements in glycaemic control and weight for 2 years as monotherapy compared with glimepiride in patients with type 2 diabetes

**DOI:** 10.1111/j.1463-1326.2010.01356.x

**Published:** 2011-04

**Authors:** A Garber, R R Henry, R Ratner, P Hale, C T Chang, B Bode, on behalf of the LEAD-3 (Mono) Study Group

**Affiliations:** 1 Department of Medicine and the Division of Endocrinology, Diabetes and Metabolism, Baylor College of MedicineHouston, TX, USA; 2 Department of Medicine, University of California at San DiegoSan Diego, CA, USA; 3 Endocrinology and MetabolismVeteran Affairs, San Diego, CA, USA; 4 MedStar Health Research InstituteHyattsville, MD, USA; 5 Novo Nordisk, Inc.Princeton, NJ, USA; 6 Atlanta Diabetes AssociatesAtlanta, GA, USA

**Keywords:** dipeptidyl peptidase-4, exenatide, glimepiride, GLP-1 analogue, incretins

## Abstract

**Aims:** Most treatments for type 2 diabetes fail over time, necessitating combination therapy. We investigated the safety, tolerability and efficacy of liraglutide monotherapy compared with glimepiride monotherapy over 2 years.

**Methods:** Participants were randomized to receive once-daily liraglutide 1.2 mg, liraglutide 1.8 mg or glimepiride 8 mg. Participants completing the 1-year randomized, double-blind, double-dummy period could continue open-label treatment for an additional year. Safety data were evaluated for the full population exposed to treatment, and efficacy data were evaluated for the full intention-to-treat (ITT) and 2-year completer populations. Outcome measures included change in glycosylated haemoglobin (HbA1c), fasting plasma glucose (FPG), body weight and frequency of nausea and hypoglycaemia.

**Results:** For patients completing 2 years of therapy, HbA1c reductions were −0.6% with glimepiride versus −0.9% with liraglutide 1.2 mg (difference: −0.37, 95% CI: −0.71 to −0.02; p = 0.0376) and −1.1% with liraglutide 1.8 mg (difference: −0.55, 95% CI: −0.88 to −0.21; p = 0.0016). In the ITT population, HbA1c reductions were −0.3% with glimepiride versus −0.6% with liraglutide 1.2 mg (difference: −0.31, 95% CI: −0.54 to −0.08; p = 0.0076) and −0.9% with liraglutide 1.8 mg (difference: −0.60, 95% CI: −0.83 to −0.38; p < 0.0001). For both ITT and completer populations, liraglutide was more effective in reducing HbA1c, FPG and weight. Over 2 years, rates of minor hypoglycaemia [self-treated plasma glucose <3.1 mmol/l (<56 mg/dl)] were significantly lower with liraglutide 1.2 mg and 1.8 mg compared with glimepiride (p < 0.0001).

**Conclusion:** Liraglutide monotherapy for 2 years provides significant and sustained improvements in glycaemic control and body weight compared with glimepiride monotherapy, at a lower risk of hypoglycaemia.

## Introduction

As monotherapy, most antidiabetic agents fail to maintain long-term glycaemic control. Although at different rates, this has been shown in the UK Prospective Diabetes Study (UKPDS) [1] and A Diabetes Outcome Progression Trial (ADOPT) [2] with either metformin, sulphonylurea (SU), or thiazolidinedione (TZD) monotherapy.

Liraglutide is a once-daily glucagon-like peptide 1 (GLP-1) analogue for treatment of type 2 diabetes with 97% homology to human GLP-1. It is safe and effective when used alone or in combination with metformin [3–5], an SU [4,6], metformin + TZD [7] or metformin + SU [4,8]. In the 52-week Liraglutide Effect and Action in Diabetes (LEAD)-3 trial [9], liraglutide monotherapy produced greater reductions in glycosylated haemoglobin (HbA1c) (primary endpoint), fasting plasma glucose (FPG), body weight and systolic blood pressure (SBP) than glimepiride 8 mg monotherapy, with fewer episodes of hypoglycaemia. At the 1.8 mg dose, liraglutide produced stable glycaemic control, with HbA1c levels unchanged at 52 weeks, as compared to the 12-week nadir.

The objective of this 52-week open-label LEAD-3 extension was not only to evaluate the long-term (2-year) safety, tolerability and efficacy of liraglutide monotherapy compared with glimepiride monotherapy, but also to assess durability of glycaemic control, using various analytical procedures.

## Research Design and Methods

### Participants and Trial Design

LEAD-3 was a 104-week, phase 3, multicentre (126 US and 12 Mexican sites), active-control, parallel-group trial with an initial 52-week randomized, double-blind, double-dummy treatment period [9] followed by a 52-week open-label extension. Participants with type 2 diabetes [18–80 years, body mass index ≤45 kg/m^2^, screening HbA1c 7–11% (53–97 mmol/mol) on diet/exercise or 7–10% (53–86 mmol/mol) on oral antidiabetic drug monotherapy] were randomized (1 : 1 : 1) to receive once-daily subcutaneous liraglutide 1.2 mg, liraglutide 1.8 mg or oral glimepiride 8 mg. Participants remained in their original randomized treatment group with no dose changes during the extension. Inclusion/exclusion and withdrawal criteria have been reported previously [9]. Participants with three consecutive FPG values >13.3 mmol/l (240 mg/dl) after week 8 and >12.2 mmol/l (220 mg/dl) after week 28, or who did not achieve adequate glycaemic control in the opinion of the investigator, were withdrawn for ‘ineffective therapy’.

The trial was conducted from 7 February 2006 to 10 November 2008. Participants were unblinded to treatment allocation at their first visit after the 1-year database release (22 November 2007). This trial is registered with ClinicalTrials.gov (NCTC00294723) and was conducted in compliance with Declaration of Helsinki and Good Clinical Practice guidelines [10]. All amendments were approved by local institutional review boards. Participants provided written informed consent prior to any trial activities and continuation in the extension.

The primary endpoint of the LEAD-3 trial was the change in HbA1c from baseline to week 52. All endpoints for the extension were secondary endpoints, with the key extension efficacy variable being the change in HbA1c from baseline to week 104. Secondary efficacy variables, described in the previous report [body weight, waist circumference, FPG, mean postprandial plasma glucose (PPG) from eight-point self-measured plasma glucose profiles, SBP, homeostasis model assessment of *β*-cell function (HOMA-B), homeostasis model assessment of insulin resistance (HOMA-IR), pro-insulin to insulin ratio, and fasting glucagon, insulin and C-peptide] were also evaluated at 104 weeks. Safety assessments were identical to those reported previously and key assessments included adverse events, hypoglycaemia and calcitonin [9].

### Statistical Analyses

**Figure 1:**
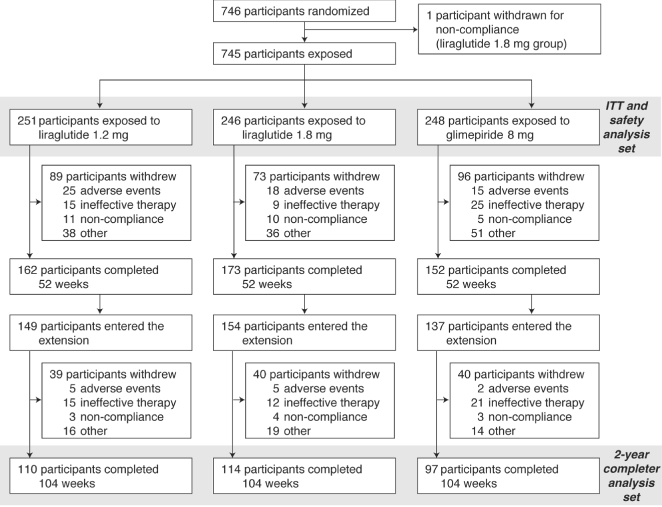
Participant flow during the LEAD-3 trial. ITT, intention-to-treat.

As extension efficacy results can be influenced by choice of analysis set and types of statistical evaluations, we analysed two different populations (figure 1), intention-to-treat (ITT) and completers, and used various statistical methods to handle missing efficacy data. Between-treatment-group comparisons of efficacy outcomes were analysed by analysis of covariance (ancova) with treatment, country, and previous antidiabetes treatment as fixed effects and baseline as covariate. ancova analyses of completers did not have imputation. For ITT analyses, missing values were imputed using last observation carried forward (LOCF), enabling all data (even from participants who withdrew early) to be analysed at week 104. In addition, post-baseline estimates and treatment comparisons were obtained using repeated-measures ancova [Proc Mixed procedure with repeated statement from sas version 9.1.3 (sas Institute, Cary, NC, USA)]. This robust model reduced variability at each post-baseline timepoint by taking into account the overall trend of data for each treatment group; significance (p < 0.05) was determined for each post-baseline timepoint without adjusting the significance level. National Glycohemoglobin Standardization Program (NGSP) HbA1c values (in %) were converted into International Federation of Clinical Chemistry (IFCC) values (in mmol/mol) using the master equation: IFCC = 10.93 NGSP − 23.50.

The full safety analysis set (all participants exposed to treatment) was used for all safety outcomes. Hypoglycaemia event rates were compared using a generalized linear model with treatment and country as fixed effects under negative binomial distribution. Other safety outcomes and demographic data were presented using descriptive statistics. Cumulative adverse events over 2 years are reported.

### Funding Source and Role of the Sponsor

Novo Nordisk sponsored this trial and contributed to protocol design, statistical analysis plans for 1-year and 2-year data, data management, statistical analyses, and reporting of results. The authors participated in trial design and had full access to both 1-year and 2-year data. The authors participated in writing and editing manuscript drafts, and assume full responsibility for the data reported herein; authors made the final decision to submit this manuscript for publication.

## Results

As shown in figure 1, 487/746 (65%) randomized participants completed 1 year of double-blinded treatment, 440/487 (90%) continued in the extension and 321/440 (73%) completed 2 years (43% of the original randomized population). Of all randomized participants, fewer withdrew during year 2 (119/746, 16%) than during year 1 (259/746, 35%). Withdrawals for various reasons for year 2 were comparable between groups (figure 1). During the second year, the most common reason for trial withdrawal was ‘other’ (e.g. participants who withdrew consent, were lost to follow-up, or moved) in the liraglutide groups and ‘ineffective therapy’ in the glimepiride group (figure 1). Demographic characteristics for randomized participants and 2-year completers were similar overall, but 2-year completers had slightly lower mean HbA1c, FPG and duration of diabetes (Table 1).

**Table 1:** Participant demographics and baseline (at randomization) characteristics

	Liraglutide 1.2 mg		Liraglutide 1.8 mg		Glimepiride 8 mg	
			
	All randomized	2-year completers	All randomized	2-year completers	All randomized	2-year completers
N	251	110	247	114	248	97
Men, n (%)	117 (47)	48 (44)	121 (49)	56 (49)	133 (54)	60 (62)
Age, years	53.7 (11.0)	53.8 (10.5)	52.0 (10.8)	52.8 (8.8)	53.4 (10.9)	54.0 (9.7)
Race, n (%)						
White	200 (80)	94 (85)	186 (75)	86 (75)	192 (77)	80 (82)
Black	34 (14)	7 (6)	30 (12)	16 (14)	30 (12)	8 (8)
Asian	5 (2)	2 (2)	12 (5)	3 (3)	9 (4)	1 (1)
Other	12 (5)	7 (6)	19 (8)	9 (8)	17 (7)	8 (8)
Hispanic ethnicity, n (%)	81 (32)	43 (39)	87 (35)	44 (39)	93 (38)	50 (52)
BMI, kg/m^2^	33.2 (5.6)	33.2 (5.5)	32.8 (6.3)	33.0 (6.4)	33.2 (5.6)	32.5 (5.6)
Duration diabetes, years	5.2 (5.5)	4.4 (5.5)	5.3 (5.1)	4.5 (4.5)	5.6 (5.1)	4.9 (4.7)
Previous treatment, n (%)						
Diet/exercise	91 (36)	41 (37)	87 (35)	43 (38)	94 (38)	33 (34)
OAD monotherapy	160 (64)	69 (63)	160 (65)	71 (62)	154 (62)	64 (66)
HbA1c, %*	8.2 (1.1)	8.0 (1.0)	8.2 (1.1)	8.1 (1.0)	8.2 (1.1)	8.0 (1.0)
Weight, kg	92.1 (19.0)	92.0 (19.1)	92.6 (20.8)	92.6 (20.8)	93.3 (19.0)	90.5 (17.5)
FPG, mmol/l	9.3 (2.6)	8.7 (2.1)	9.5 (2.6)	9.1 (2.2)	9.5 (2.6)	8.7 (2.1)
SBP, mmHg	127.6 (14.3)	125.2 (12.0)	128.0 (13.9)	128.6 (14.7)	130.0 (16.1)	129.5 (16.7)
HOMA-B, %	65.6 (50.2)	72.2 (50.3)	65.5 (62.7)	74.4 (75.1)	74.9 (89.6)	74.8 (71.2)
HOMA-IR, %	7.0 (6.7)	6.4 (3.8)	6.9 (5.6)	6.9 (5.8)	7.5 (6.2)	6.6 (4.4)

Data are mean (s.d.) unless otherwise noted. HbA1c, weight, FPG and SBP values are from randomization (week 0). All other values are from screening. BMI, body mass index; FPG, fasting plasma glucose; HbA1c, glycosylated haemoglobin; HOMA-B, homeostasis model assessment of *β*-cell function; HOMA-IR, homeostasis model assessment of insulin resistance; OAD, oral antidiabetic drug; SBP, systolic blood pressure.

* HbA1c values of 8.0, 8.1 and 8.2% are equivalent to 64, 65 and 66 mmol/mol, respectively.

**Figure 2:**
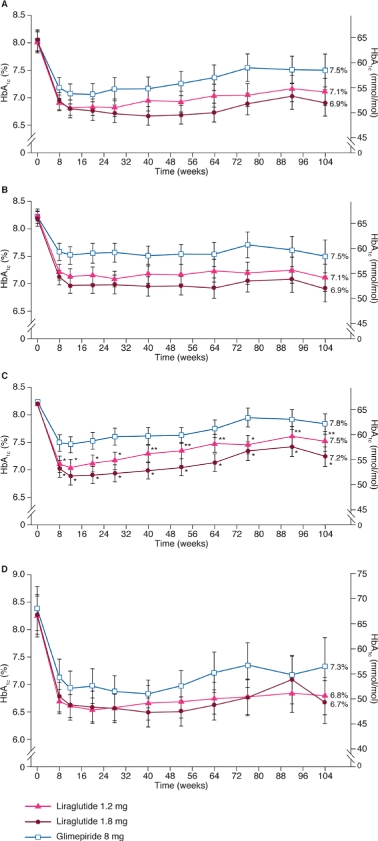
HbA1c over time. (A) Two-year completer population, observed mean values, no imputation. (B) Intention-to-treat (ITT) population, observed mean values, no imputation. (C) ITT population, estimated least square (LS) mean values derived from an analysis of covariance (ancova) model with repeated measures. (D) Two-year completer population, participants previously treated with diet and exercise, observed mean values, no imputation. Error bars are ±2s.e. Panel C: ^*^p < 0.0001 vs. glimepiride; ^**^p < 0.05 vs. glimepiride.

As extension trials tend to show survivor bias and to ensure that potential bias was minimized, two populations were evaluated: 2-year completers and the full ITT population. Mean HbA1c over time was plotted for 2-year completers (observed values, no imputation) in figure 2A; using ancova with no imputation, estimated HbA1c reductions from baseline to 2 years were significantly greater with liraglutide 1.2 mg [−0.9%; estimated treatment difference (ETD): −0.37, 95% CI: −0.71 to −0.02; p = 0.0376] and liraglutide 1.8 mg (−1.1%; ETD: −0.55, 95% CI: −0.88 to −0.21; p = 0.0016 ) compared with glimepiride (−0.6%).

Mean HbA1c over 2 years is plotted for the ITT population in figure 2B (observed values, no imputation); using ancova with LOCF imputation, estimated HbA1c reductions from baseline to 2 years were significantly greater with liraglutide 1.2 mg (−0.6%; ETD: −0.31, 95% CI: −0.54 to −0.08; p = 0.0076) and liraglutide 1.8 mg (−0.9%; ETD: −0.60, 95% CI: −0.83 to −0.38; p < 0.0001) compared with glimepiride (−0.3%). Although the HbA1c reductions from baseline were smaller in the ITT (LOCF) analysis set compared with the 2-year completer analysis set, ETDs were very similar. In addition, estimated values of HbA1c from a repeated-measures ancova were significantly different between liraglutide and glimepiride at all post-baseline timepoints (figure 2C, ITT population). Thirty-six percent of 2-year completers were drug-naive at trial entry; mean HbA1c values over 2 years are presented in figure 2D.

**Figure 3:**
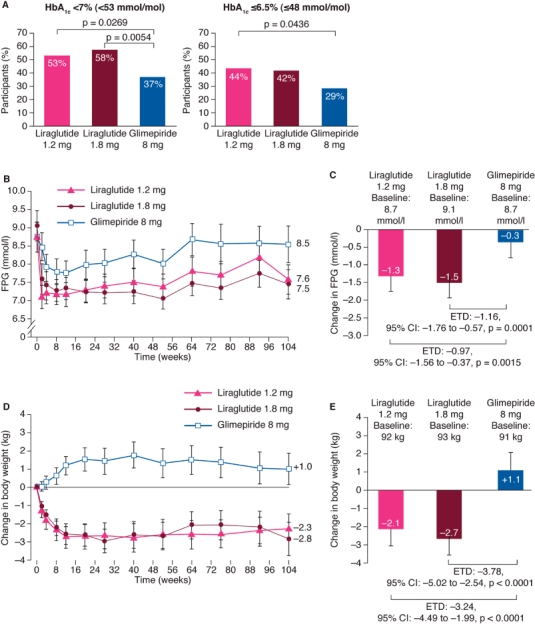
Additional efficacy endpoints for 2-year completers. (A) Percentage of participants treated to HbA1c targets <7% (<53 mmol/mol) (left graph) and ≤6.5% (≤48 mmol/mol) (right graph) at 2 years. (B) Fasting plasma glucose (FPG) (mmol/l) over time. (C) Change in FPG (mmol/l) from baseline to week 104. (D) Body weight (kg) over time. (E) Change in body weight (kg) from baseline to week 104. Data in panel A are estimated percentages from a logistic regression model. Data in panels B and D are observed means. Data in panels C and E are least square (LS) means from an analysis of covariance (ancova) model. Error bars are ±2s.e. ETD = estimated treatment difference (liraglutide–glimepiride).

More 2-year completers treated with liraglutide reached the American Diabetes Association (ADA) HbA1c target of <7% (<53 mmol/mol) and the more stringent International Diabetes Federation (IDF)/American Association of Clinical Endocrinologists (AACE) target of ≤6.5% (≤48 mmol/mol) compared with glimepiride (figure 3A). In 2-year completers, FPG declined early in the trial and these decreases were maintained (figure 3B), and liraglutide was significantly more effective than glimepiride in reducing FPG (figure 3C). Reductions in mean PPG were −1.9, −2.6 and −1.8 mmol/l for liraglutide 1.2 mg, liraglutide 1.8 mg and glimepiride (p = 0.0103 for liraglutide 1.8 mg vs. glimepiride).

In 2-year completers, mean body weight decreased over the first 12 weeks of therapy and decreases were maintained over 2 years in both liraglutide groups; the glimepiride group gained weight (figure 3D). Differences between treatment groups were statistically significant (figure 3E). Participants also had significant reductions in waist circumference with liraglutide 1.2 mg (4.0 cm) and liraglutide 1.8 mg (4.9 cm) compared with glimepiride (1.0 cm) (p = 0.0413 for liraglutide 1.2 mg and p = 0.0095 for liraglutide 1.8 mg vs. glimepiride).

In 2-year completers, HOMA-IR decreased by 1.1% with liraglutide 1.2 mg and 0.8% with liraglutide 1.8 mg and increased by 0.8% with glimepiride (p = 0.0451 for liraglutide 1.2 mg vs. glimepiride). Pro-insulin to insulin ratio increased slightly in all groups, by 0.108 with liraglutide 1.2 mg, 0.018 with liraglutide 1.8 mg and 0.141 with glimepiride (with significantly less of an increase with liraglutide 1.8 mg vs. glimepiride; p = 0.0394). After 2 years, all three groups had increases in HOMA-B, fasting insulin and fasting C-peptide, and had decreases in fasting glucagon, but the differences between groups were not significant. No significant differences between groups in change in pulse, diastolic blood pressure (DBP) and SBP were observed in participants completing 2 years. Although the SBP differences were not significant at week 104, there were significantly greater reductions with liraglutide versus glimepiride at the majority of timepoints [weeks 2, 4, 8, 12, 20, 40, 76 (1.8 mg only) and 92] using a repeated-measures ancova analysis.

Besides HbA1c, all other efficacy endpoints were also analysed using the full ITT analysis set (ancova, LOCF) (Table 2). Estimated mean treatment differences for the ITT population were similar to 2-year completers with significantly greater reductions in FPG, body weight, waist circumference and HOMA-IR, and significantly greater increases in proportion of participants reaching HbA1c targets of <7% (<53 mmol/mol) and ≤6.5% (≤48 mmol/mol) for liraglutide 1.2 mg and 1.8 mg compared with glimepiride. In contrast to the 2-year completer population, the ITT population had significant reductions with liraglutide 1.2 mg compared with glimepiride in fasting glucagon and with both liraglutide 1.2 mg and 1.8 mg compared with glimepiride in HOMA-IR. As with completers, there were no significant differences between treatment groups in SBP, DBP, pulse and HOMA-B after 2 years in the ITT population.

**Table 2:** Change from baseline to week 104 for secondary efficacy parameters [intention-to-treat (ITT) Population]

	Liraglutide 1.2 mg		Liraglutide 1.8 mg		Glimepiride 8 mg
			
Endpoint	Δ	ETD [95% CI] and p-value versus glimepiride	Δ	ETD [95% CI] and p-value versus glimepiride	Δ
HbA1c, %	−0.6	−0.31 [−0.54; −0.08], **0.0076**	−0.9	−0.60 [−0.83; −0.38], **<0.0001**	−0.3
HbA1c <7% (<53 mmol/mol), % participants*	36.9	1.98 [1.30; 3.00], **0.0015**	44.4	2.81 [1.85; 4.26], **<0.0001**	23.2
HbA1c ≤6.5%, (<48 mmol/mol) % participants*	25.0	1.84 [1.15; 2.95], **0.0113**	29.9	2.39 [1.50; 3.80], **0.0002**	15.4
FPG, mmol/l	−0.52	−0.63 [−1.17; −0.09], **0.0217**	−0.88	−0.99 [−1.53; −0.45], **0.0003**	0.11
PPG, mmol/l	−1.52	−0.14 [−0.66; 0.39], 0.6060	−2.06	−0.68 [−1.21; −0.16], **0.0105**	−1.38
Body weight, kg	−1.89	−2.84 [−3.63; −2.06], **<0.0001**	−2.70	−3.65 [−4.44; −2.86], **<0.0001**	0.95
Waist circumference, cm	−3.48	−3.08 [−4.72; −1.44], **0.0002**	−3.73	−3.33 [−4.98; −1.69], **<0.0001**	−0.40
HOMA-B, %	20.63	3.72 [−21.91; 29.34], 0.7757	24.66	7.74 [−18.10; 33.58], 0.5565	16.91
HOMA-IR, %	−0.38	−1.50 [−2.92; −0.08], **0.0388**	−0.86	−1.99 [−3.42; −0.55], **0.0067**	1.13
Fasting insulin, pmol/l	−2.03	−17.91 [−41.65; 5.83], 0.1390	−1.86	−17.74 [−41.71; 6.24], 0.1468	15.88
Fasting C-peptide, nmol/l	10.05	−0.51 [−9.47; 8.46], 0.9117	14.36	3.80 [−5.27; 12.87], 0.4104	10.56
Pro-insulin/insulin ratio	0.05	−0.05 [−0.12; 0.02], 0.1403	0.01	−0.10 [−0.17; −0.03], **0.0045**	0.10
Fasting glucagon, pg/ml	−11.85	−6.86 [−13.52; −0.21], **0.0433**	−9.92	−4.93 [−11.69; 1.84], 0.1535	−4.99
SBP, mmHg	−1.35	−0.86 [−3.18; 1.46], 0.4657	−2.37	−1.88 [−4.21; 0.45], 0.1135	−0.49
DBP, mmHg	−0.58	−0.14 [−1.50; 1.23], 0.8429	−0.81	−0.37 [−1.74; 1.00], 0.5965	−0.44
Pulse, bpm	2.04	1.36 [−0.17; 2.90], 0.0821	0.92	0.24 [−1.30; 1.78], 0.7589	0.67

Δ = Change from baseline to the end of the trial (week 104), ITT (last observation carried forward). Data are least square means, unless otherwise noted. p-values from statistically significant comparisons are in bold text. bpm, beats per minute; CI, confidence interval; DBP, diastolic blood pressure; ETD, estimated treatment difference (liraglutide–glimepiride); FPG, fasting plasma glucose; HbA1c, glycosylated haemoglobin; HOMA-B, homeostasis model assessment of *β*-cell function; HOMA-IR, homeostasis model assessment of insulin resistance; PPG, postprandial plasma glucose; SBP, systolic blood pressure.

* Percentage reaching target from a logistic regression model and odds ratio presented rather than treatment difference.

Adverse events (system organ classes and preferred terms) reported by at least 5% of participants during the 2-year trial period are summarized for the entire safety population; gastrointestinal adverse events were more common with liraglutide (Table 3). With liraglutide, nausea was most frequently reported early in the trial and remained below 5% throughout the extension. No participants withdrew from the extension because of nausea.

**Table 3:** Treatment-emergent adverse events over 2 years by system organ class and preferred term reported by ≥5% in any one group (safety population)

	Liraglutide 1.2 mg (N = 251)	Liraglutide 1.8 mg (N = 246)	Glimepiride 8 mg (N = 248)
All adverse events	213(85%)	207(84%)	194(78%)
Gastrointestinal disorders	135(54%)	130(53%)	70(28%)
Constipation	21(8%)	29(12%)	12(5%)
Diarrhoea	44(18%)	48(20%)	23(9%)
Flatulence	4(2%)	13(5%)	5(2%)
Nausea	72(29%)	75(31%)	21(9%)
Vomiting	33(13%)	25(10%)	10(4%)
Cardiac disorders	8(3%)	11(5%)	14(6%)
Eye disorders	15(6%)	14(6%)	16(7%)
General disorders and administration site conditions	42(17%)	51(21%)	39(16%)
Fatigue	8(3%)	13(5%)	9(4%)
Infections and infestations	134(53%)	124(50%)	114(46%)
Bronchitis	15(6%)	9(4%)	11(4%)
Influenza	23(9%)	27(11%)	21(9%)
Nasopharyngitis	23(9%)	16(7%)	18(7%)
Sinusitis	21(8%)	18(7%)	18(7%)
Upper respiratory tract infection	36(14%)	33(13%)	22(9%)
Urinary tract infection	26(10%)	15(6%)	13(5%)
Injury, poisoning and procedural complications	32(13%)	35(14%)	37(15%)
Investigations	23(9%)	31(13%)	25(10%)
Metabolism and nutrition disorders	45(18%)	42(17%)	36(15%)
Musculoskeletal, connective tissue disorders	61(24%)	61(25%)	58(23%)
Arthralgia	11(4%)	6(2%)	15(6%)
Back pain	18(7%)	18(7%)	17(7%)
Pain in extremity	9(4%)	15(6%)	8(3%)
Nervous system disorders	65(26%)	58(24%)	58(23%)
Dizziness	13(5%)	19(8%)	13(5%)
Headache	28(11%)	18(7%)	23(9%)
Psychiatric disorders	24(10%)	28(11%)	16(7%)
Depression	8(3%)	14(6%)	5(2%)
Renal and urinary disorders	13(5%)	11(5%)	15(6%)
Reproductive system and breast disorders	12(5%)	14(6%)	12(5%)
Respiratory, thoracic and mediastinal disorders	23(9%)	42(17%)	36(15%)
Cough	5(2%)	14(6%)	11(4%)
Skin and subcutaneous tissue disorders	26(10%)	31(13%)	20(8%)
Vascular disorders	20(8%)	20(8%)	20(8%)
Hypertension	14(6%)	11(5%)	17(7%)

Data are n (%).

Over 2 years, 28 serious adverse events were reported by 23 (9.2%) participants using liraglutide 1.2 mg, 30 events by 22 (8.9%) participants using liraglutide 1.8 mg and 32 events by 20 (8.1%) participants using glimepiride. Most events were ‘unlikely’ related to trial drug with 12 serious adverse events judged to have a ‘possible’ relationship (Appendix S2). During the extension, there was 1 fatality after 669 days of treatment with liraglutide 1.8 mg. The participant died at home 3 days after a colonoscopy and biopsy of a polyp; autopsy revealed signs of acute and chronic pancreatitis and cholelithiasis. No amylase or lipase measurements were available.

Over 2 years, 12, 10 and 26% of participants had minor hypoglycaemia [self-treated plasma glucose <3.1 mmol/l (56 mg/dl)] in the liraglutide 1.2 mg, liraglutide 1.8 mg and glimepiride groups, respectively. The rate of minor hypoglycaemia was 0.21 events per patient-year with liraglutide 1.2 mg and 0.22 with liraglutide 1.8 mg, significantly lower than 1.75 with glimepiride (p < 0.0001 for both comparisons vs. glimepiride). One event of major hypoglycaemia (liraglutide 1.8 mg group) occurred after regular insulin was infused as part of a substudy procedure. No increases in mean calcitonin levels were observed over 2 years and all mean values remained in the lower end of the normal range (<1.1 pg/ml).

## Discussion

Long-term head-to-head comparative studies of antidiabetes monotherapies are rare. Two-year results from published head-to-head studies comparing antidiabetic monotherapies showed greater HbA1c reductions with rosiglitazone 8 mg and metformin 2 g than glyburide 15 mg in ADOPT [2] and significantly greater reductions in HbA1c with once-daily rosiglitazone 8 mg compared with twice-daily vildagliptin 50 mg [11]. While there have been no published results from long-term controlled studies of GLP-1 receptor agonists as monotherapy, pooled 2-year results from three open-label, uncontrolled extensions of exenatide added on to metformin, SU, or both have been published and showed improvements in glycaemic control and weight [12]. Our trial, LEAD-3, was designed as a parallel-group, head-to-head monotherapy trial evaluating the 2-year safety and efficacy of liraglutide (1.2 and 1.8 mg) compared with glimepiride 8 mg. Over 2 years, liraglutide monotherapy provided significantly greater improvements in glycaemic control and body weight compared with glimepiride monotherapy, at a lower risk of hypoglycaemia.

With regard to the trial design of LEAD-3, one difference from prior long-term monotherapy trials [2,11] was that only one third of participants in LEAD-3 were previously drug-naive. Hence, substitution of liraglutide for another antidiabetes therapy could underestimate the treatment effect compared with studies where all participants were drug-naive. Indeed, larger HbA1c reductions occurred in the previously drug-naive subgroup (−1.4% in 2-year completers) compared with the entire trial population in LEAD-3. During the LEAD-3 extension, unlike the exenatide extensions [12], participants remained in their original randomized treatment group and drug doses remained unchanged. Moreover, no rescue medications were permitted. This allowed long-term comparative efficacy and safety to be assessed without these confounding factors. Limitations of this trial included the open-label nature of the extension, which can result in investigator and/or participant bias, and the 57% withdrawal rate over 2 years.

We analysed and reported efficacy outcomes both for all participants completing 2 years of therapy and the original ITT population, because efficacy results from clinical trial extensions can be influenced by the choice of analysis set. Arguably, the most appropriate analysis set from a clinical perspective would be the participants who completed the trial, which would show the population of participants that is most likely to tolerate a given therapy and what long-term efficacy and safety can be expected. Analyses of the entire trial population (ITT or safety) give an overall picture of the comparative efficacy and safety between two treatments; these are often preferred by regulatory authorities [13].

Not surprisingly, some survivor bias was observed, in that HbA1c reductions were greater for participants completing the full 2 years than in the full ITT randomized population (using no imputation for completers vs. LOCF for the ITT population). This difference may arise from the inclusion of data from participants who withdrew from the trial because of ineffective therapy, non-compliance with the protocol, adverse events, or other reasons. Participants who completed the trial had greater initial reductions in glycaemic control and had slightly lower HbA1c and FPG values at baseline. Despite the small baseline differences between the ITT and completer populations, the 2-year data were robust. Various populations and analyses showed significant and consistent treatment benefits of all three interventions, which were greater with liraglutide compared with glimepiride.

For participants remaining in the trial (43% of randomized participants), HbA1c levels after 2 years were similar to the values after 1 year, providing evidence of sustained glycaemic benefit of liraglutide therapy for at least 2 years and superiority of liraglutide as compared with glimepiride. These conclusions are robust because each method of analysis yielded essentially the same result. Moreover, participants treated with liraglutide experienced greater and sustained weight loss than those treated with glimepiride, who gained weight.

With regard to safety, in the entire safety population, the frequency of nausea with liraglutide remained low during the extension, consistent with the conclusion made after the first year that nausea is transient with liraglutide. The rates of minor hypoglycaemia associated with liraglutide were slightly lower at year 2 than year 1 (0.21–0.22 vs. 0.30–0.25 events per patient-year) [9]. Over 2 years, the risk of minor hypoglycaemia was significantly lower with liraglutide than glimepiride, consistent with GLP-1 receptor agonists stimulating insulin secretion in a glucose-dependent manner (i.e. only during hyperglycaemia) compared with SUs stimulating secretion of insulin at any blood glucose concentration. The frequency of withdrawal during the second year of the trial was lower than during the first year, and no additional safety signals were evident. Data concerning potential thyroid cancer effects of chronic liraglutide administration were reassuring. Calcitonin levels for the full 2-year period were far below the lower limits of normal for the assay. Upward drift in calcitonin levels was not seen in any treatment group at any time interval.

In conclusion, glycaemic control, weight benefits, and a low rate of hypoglycaemia were observed after 2 years of liraglutide treatment. Longer-term data are needed to confirm durability, impact upon *β*-cell function, and safety.

## Supporting Information

**Appendix S1.** LEAD-3 principal investigators.

**Appendix S2.** Treatment-emergent serious adverse events over 2 years judged to have a possible relationship to trial drug by the investigator (safety population).

## References

[1] UKPDS Group (1998). Intensive blood-glucose control with sulphonylureas or insulin compared with conventional treatment and risk of complications in patients with type 2 diabetes (UKPDS 33). UK Prospective Diabetes Study (UKPDS) Group.. Lancet.

[2] Kahn SE, Haffner SM, Heise MA (2006). Glycemic durability of rosiglitazone, metformin, or glyburide monotherapy.. N Engl J Med.

[3] Nauck M, Frid A, Hermansen K (2009). Efficacy and safety comparison of liraglutide, glimepiride, and placebo, all in combination with metformin, in type 2 diabetes: the LEAD (liraglutide effect and action in diabetes)-2 study.. Diabetes Care.

[4] Buse JB, Rosenstock J, Sesti G (2009). Liraglutide once a day versus exenatide twice a day for type 2 diabetes: a 26-week randomised, parallel-group, multinational, open-label trial (LEAD-6).. Lancet.

[5] Pratley RE, Nauck M, Bailey T, 1860-LIRA-DPP-4 Study Group (2010). Liraglutide versus sitagliptin for patients with type 2 diabetes who did not have adequate glycaemic control with metformin: a 26-week, randomised, parallel-group, open-label trial.. Lancet.

[6] Marre M, Shaw J, Brandle M (2009). Liraglutide, a once-daily human GLP-1 analogue, added to a sulphonylurea over 26 weeks produces greater improvements in glycaemic and weight control compared with adding rosiglitazone or placebo in subjects with Type 2 diabetes (LEAD-1 SU).. Diabet Med.

[7] Zinman B, Gerich J, Buse JB (2009). Efficacy and safety of the human glucagon-like peptide-1 analog liraglutide in combination with metformin and thiazolidinedione in patients with type 2 diabetes (LEAD-4 Met+TZD).. Diabetes Care.

[8] Russell-Jones D, Vaag A, Schmitz O (2009). Liraglutide vs insulin glargine and placebo in combination with metformin and sulfonylurea therapy in type 2 diabetes mellitus (LEAD-5 met+SU): a randomised controlled trial.. Diabetologia.

[9] Garber A, Henry R, Ratner R (2009). Liraglutide versus glimepiride monotherapy for type 2 diabetes (LEAD-3 Mono): a randomised, 52-week, phase III, double-blind, parallel-treatment trial.. Lancet.

[10] International Conference on Harmonisation (1996). http://www.ich.org/LOB/media/MEDIA482.pdf.

[11] Rosenstock J, Niggli M, Maldonado-Lutomirsky M (2009). Long-term 2-year safety and efficacy of vildagliptin compared with rosiglitazone in drug-naive patients with type 2 diabetes mellitus.. Diabetes Obes Metab.

[12] Buse JB, Klonoff DC, Nielsen LL (2007). Metabolic effects of two years of exenatide treatment on diabetes, obesity, and hepatic biomarkers in patients with type 2 diabetes: an interim analysis of data from the open-label, uncontrolled extension of three double-blind, placebo-controlled trials.. Clin Ther.

[13] International Conference on Harmonisation (1998). http://www.ich.org/cache/compo/475-272-1.html#E9.

