# Supplementary material for: Liraglutide, a once-daily human glucagon-like peptide 1 analogue, provides sustained improvements in glycaemic control and weight for 2 years as monotherapy compared with glimepiride in patients with type 2 diabetes
Source: Diabetes Obes Metab. 2011 Apr;13(4):348–56. doi: 10.1111/j.1463-1326.2010.01356.x (PMC3084519; doi:10.1111/j.1463-1326.2010.01356.x)
Supplement: Supplementary file 1 [file dom0013-0348-SD1.doc]

**Supplementary Appendix S1. LEAD-3 Principal Investigators**

Arechavaleta-Granell M, García-Hernández P, Gonzalez-Galvez G, Gonzalez-Villalpando C, Morales-Flores H, Olvera-Alvarez I, Rios-Rodriguez E, Rodriguez-Pattzi H, Salinas-Gonzalez F, Sauque-Reyna L, Sosa-Camas R, Tamez-Perez H, Violante-Ortiz R, Abbott L, Arakaki R, Bailey T, Blonde L, Bode B, Bressler P, Brusco O, Chisolm O, Rothman J, Corbett B, Cullen E, Downey H, Duckor S, Farmer H, Farrell J, Feinglos M, Fusco F, Garber A, Goldstein B, Gollapudi G, Graf R, Greco S, Hartman I, Hassman D, Mudaliar S, Hodge R, Hoffman B, Hollander P, Kaplan R, Kapoor A, Kawley F, Klein E, Landgarten S, Carroll M, Leslie H, Licata A, Linden D, Lipetz R, McGill J, Clarke D, Mezitis N, Morris L, Mulmed L, Myers L, Ollins R, Palte S, Pearlstein R, Peterson G, Phillips L, Popeil L, Powers C, Race J, Rivera-Colon L, Rosenstock J, Sharma S, Schumacher D, Schwartz S, Shelmet J, Shepherd M, Silver B, Snell P, Snyder B, Sugimoto D, Sussman A, Tamayo R, Tisovec R, Cheatham W, Wahl T, Warren M, Weinrib S, Weinstein R, Weinstock R, Weiss D, Williams R, Witkin D, Cox M, Zisser H, East H, Barrera J, Gilman R, Robinson M, Pullman J, Ajani D, Reichman A, Thigpen D, Zieve F, Fishman N, Mather K, Abelseth J, Corder C, Griffing G, Huffman D, Hunt G, Lochner J, Ratcliff L, Shomali M, Leichter S, Manlove-Simmons K, Thorne D, Garcia-Soria-Davis M, Granados Reyes S, Gallen J, Furlong K, Uwaifo G, Aroda V, Wynne A, Gupta A, Oberoi M, Brunner J, Christensen R, Lucas K, Baron M, Picciano M, Prasad S, Reed L, Silver G, Dunn L, Sperling M, Sullivan J, Levinson L, Beecher A, Marple R, Rasor D, Idowu O, Khan J, Oyer D, Topkis R, Spees R, Peniston J, Ryckman G, Philis-Tsimikas A, Gaona R, Askari N, Choi L.
