# Supplementary material for: Liraglutide, a once-daily human glucagon-like peptide 1 analogue, provides sustained improvements in glycaemic control and weight for 2 years as monotherapy compared with glimepiride in patients with type 2 diabetes
Source: Diabetes Obes Metab. 2011 Apr;13(4):348–56. doi: 10.1111/j.1463-1326.2010.01356.x (PMC3084519; doi:10.1111/j.1463-1326.2010.01356.x)
Supplement: Supplementary file 2 [file dom0013-0348-SD2.doc]

**Supplementary Appendix S2.**

**Treatment-emergent serious adverse events over 2 years judged to have a possible relationship to trial drug by the investigator (safety population)**

The 12 serious adverse events judged to have a ‘possible’ relationship to trial drug were hypoaesthesia (day 267), myocardial infarction (day 367), appendicitis perforated (day 157), hypoglycaemia (day 730), and breast cancer (day 194) in the liraglutide 1.8 mg group; gastroenteritis (day 18), myocardial infarction (day 167), and one participant with thyroid disorder (diffuse C-cell hyperplasia, day 356), benign neoplasm of the thyroid gland (day 356), and thyroid cancer (papillary microcarcinoma, day 356) in the liraglutide 1.2 mg group; and myocardial infarction (day 218) and grand mal convulsion (day 103) in the glimepiride group.
